# Deliberate Establishment of Asymptomatic Bacteriuria—A Novel Strategy to Prevent Recurrent UTI

**DOI:** 10.3390/pathogens5030052

**Published:** 2016-07-29

**Authors:** Björn Wullt, Catharina Svanborg

**Affiliations:** Department of Microbiology, Immunology and Glycobiology (MIG), Institute of Laboratory Medicine, Lund University, 221 00 Lund, Sweden; catharina.svanborg@med.lu.se

**Keywords:** asymptomatic bacteriuria (ABU), therapeutic efficacy, virulence

## Abstract

We have established a novel strategy to reduce the risk for recurrent urinary tract infection (UTI), where rapidly increasing antibiotic resistance poses a major threat. Epidemiologic studies have demonstrated that asymptomatic bacteriuria (ABU) protects the host against symptomatic infections with more virulent strains. To mimic this protective effect, we deliberately establish ABU in UTI-prone patients, who are refractory to conventional therapy. The patients are inoculated with *Escherichia coli* (*E. coli*) 83972, now widely used as a prototype ABU strain. Therapeutic efficacy has been demonstrated in a placebo-controlled trial, supporting the feasibility of using *E. coli* 83972 as a tool to prevent recurrent UTI and, potentially, to outcompete antibiotic-resistant strains from the human urinary tract. In addition, the human inoculation protocol offers unique opportunities to study host-parasite interaction in vivo in the human urinary tract. Here, we review the clinical evidence for protection using this approach as well as some molecular insights into the pathogenesis of UTI that have been gained during these studies.

## 1. Background

Asymptomatic bacteriuria (ABU) is the most common form of UTI, resembling commensalism at other mucosal sites [1]. ABU is defined by asymptomatic carriage of >10^5^ bacteria/mL of urine, in two consecutive urine cultures ([2] and Nicolle et al., this issue). In population-based studies of otherwise healthy individuals, ABU occurs in about 1% of schoolgirls [3] and in 1.9%–9.5% of pregnant women [4], depending on the access to health care. The prevalence increases to about 20% in otherwise healthy individuals over the age of 70 [5,6] and reaches 23%–89% in patients with spinal cord injuries [7]. Based on studies with varied protocols and target populations, an overall frequency of 3.5% has been proposed in adult females [8].

Patients with ABU often carry the same bacterial strain for months or years, without evidence of symptoms. In a series of studies, ABU has been shown to protect the host against symptomatic UTI [9,10]. In a three-year follow-up study, Lindberg detected a significant advantage in children with long-term ABU compared to a control group where ABU was eliminated by antibiotic therapy [11]. In a later controlled trial of untreated versus treated ABU, symptomatic recurrences occurred in 15% of the treated patients and the majority had acute pyelonephritis (APN) [12]. None of the children with un-treated ABU developed symptomatic UTI [9] and there was no evidence of adverse effects, such as decreased renal concentrating capacity [3], and no damage to the kidneys by excretory urography and micturition cystourethrography [13]. As a result of these and many other studies, the current consensus in national and international UTI treatment guidelines is to leave ABU untreated in patients without risk factors, including children [14].

We have reasoned that this natural protection by ABU offers a therapeutic opportunity, provided that the safety and efficacy of ABU is recreated. The obvious target group is patients with recurrent UTI, who do not spontaneously develop protective ABU. In addition, there may be indications for wider use, not least to prevent symptomatic UTI and reduce antibiotic use. We have therefore developed a protocol for deliberate inoculation into the lower urinary tract and explored the feasibility of this approach. In our early studies, patients were inoculated with *E. coli* isolates from their own fecal flora. The bacteria persisted for up to four months without deleterious effects [15]. A routine was developed to store the patient’s own ABU strain during other infections that required antibiotic therapy, and to re-inoculate the urinary tract once the treatment period is over, thereby re-establishing ABU with the same strain [12]. To establish ABU in a more standardized way, we selected to use *E. coli* 83972. This strain establishes ABU in different human hosts and is safe for these patients. Protective efficacy has been demonstrated in a placebo-controlled trial [16] and benefits have been confirmed in several observational studies [17,18,19].

## 2. Properties of ABU Strains

*E. coli* isolates from patients with ABU differ from the strains that cause symptomatic infections. About 80% of ABU strains adhere poorly to human uroepithelial cells [20,21,22], are sensitive to killing by serum [3] and have attenuated O- and K antigens [3,23]. In contrast to APN strains, ABU strains seldom express P fimbriae or hemolysins [24,25,26]. Paradoxically, however, about 60% of ABU strains carry residues of chromosomal copies of the virulence genes found in uropathogenic *E. coli* (UPEC) strains [27]. This discrepancy between genotype and phenotype has been explained by genome sequencing, which identified attenuating mutations and deletions in the virulence genes in the ABU strain [28] (Dobrindt et al., this issue). Based on sequence data and other techniques, about 50%–60% of ABU strains appear to be attenuated UPEC strains that have undergone reductive evolution, resulting in smaller genome sizes and non-functional virulence genes [28]. The remaining ABU strains instead resemble the commensal *E. coli* population in the fecal flora [22,29].

*E. coli* 83972 was isolated during a prospective study of childhood ABU [3], from a school-aged girl who carried the strain for at least three years without symptoms from the urinary tract or evidence of renal damage. The strain was chosen for human use as it lacked detectable virulence factors and large conjugative plasmids. *E. coli* 83972 is well adapted to growth in human urine, and outcompetes uropathogenic *E. coli* strains during in vitro growth [30]. It is susceptible to the antibiotics commonly used in UTI, and carries a 1.2 kB plasmid, which facilitates identification from clinical samples. The strain has been extensively characterized, and the genome sequence has been solved.

Sequencing of the *E. coli* 83972 genome revealed a common ancestry with uropathogenic *E. coli* strains but a smaller genome size due to multiple deletions and mutations, resulting in a smaller overall genome size and the systematic inactivation of virulence genes, either by mutations or deletions [28,30,31]. For example, a large *fim* deletion and several *papG* point mutations abolish fimbrial expression and adherence [28,31]. We have also proposed that this strain adapts to the human urinary tract by undergoing reductive evolution [31] based on genome sequencing. Phenotypic characterization of a large number of ABU strains further supported a reduction in genome size and accumulation of genomic alterations, which result in specific loss of expression or decay of UPEC virulence genes [28,30]. The properties of *E. coli* 83972 have been described in greater detail by Dobrindt et al. (this volume).

### 2.1. *E. coli* 83972 and the Inoculation Protocol

**Figure 1:**
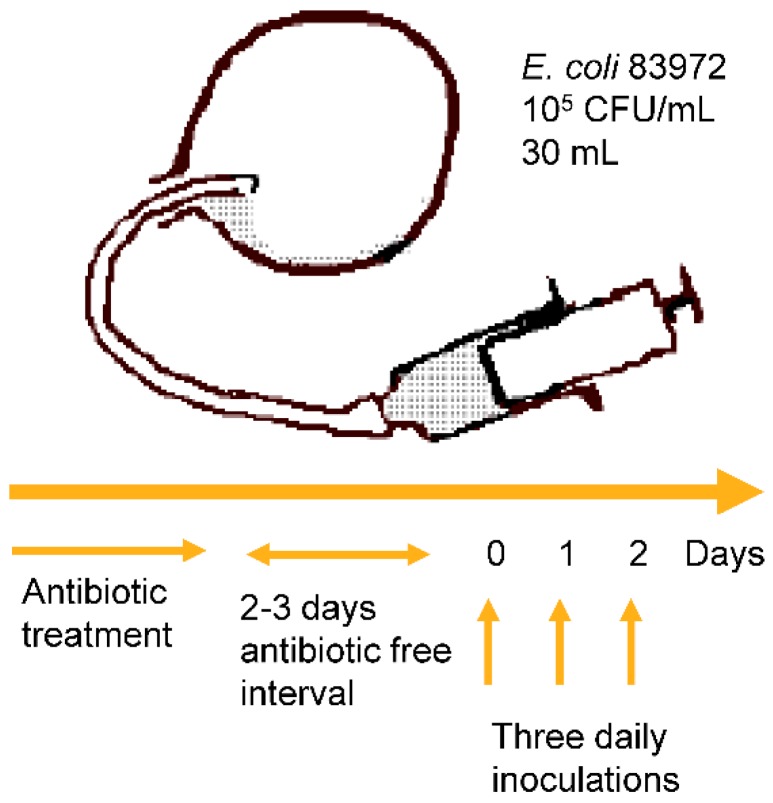
The human therapeutic inoculation protocol. Antibiotics are administered to sterilize the urine. After an antibiotic-free interval, the patient is catheterized, the bladder is emptied, and 30 mL *E. coli* 83972 (105 CFU/mL) are injected. If bacteriuria is not established, the procedure may be repeated daily for maximally three days. Published with permission from Cellular Microbiology [35].

The human urinary tract inoculation protocol is standardized and the outcome is reproducible in individual hosts [19,32]. First, appropriate antibiotics are given to sterilize the urine and after an antibiotic-free interval the patient is catheterized, and the bladder is emptied. Subsequently, 30 mL of *E. coli* 83972 (10^5^ CFU/mL) is instilled in the bladder, and the catheter is removed. Depending on the outcome, the procedure may be repeated for three days (Figure 1). The establishment of bacteriuria is defined by daily urine cultures during the first week, weekly urine cultures during the next month, and later at monthly intervals [17,33,34].

### 2.2. *E. coli* 83972 Inoculation Protects against Symptomatic UTI

Early observational studies proved the safety of the inoculation procedure [19,32,33]. These studies also showed that the outcome of inoculation depends on urodynamic factors. As expected, *E. coli* 83972, which fails to adhere to bladder epithelial cells, did not colonize patients with complete bladder emptying. *E. coli* 83972 bacteriuria was monitored for more than 600 months in 24 patients who achieved long-term colonization. In these patients, bacterial concentrations remained at >10^5^ CFU/mL, often increasing to 10^8^ CFU/mL. All patients reported subjective improvement with a reduction of minor lower urinary tract symptoms. No febrile UTI episodes were diagnosed during *E. coli* 83972 bacteriuria [34], and no side effects were registered.

The results from these observational studies encouraged us to design a double-blinded randomized trial with cross-over design. We included 20 patients with incomplete bladder emptying and/or neurogenic bladder disorders, and with a history of recurrent UTI refractory to conventional treatment. All patients were inoculated with *E. coli* 83972 or placebo and followed for 12 months, when they were crossed-over and followed for another 12 months. The study end points were A) the time to the first symptomatic infection causing UTI in both study arms, and B) the total number of symptomatic infections over the entire study period of 12 + 12 months.

**Figure 2:**
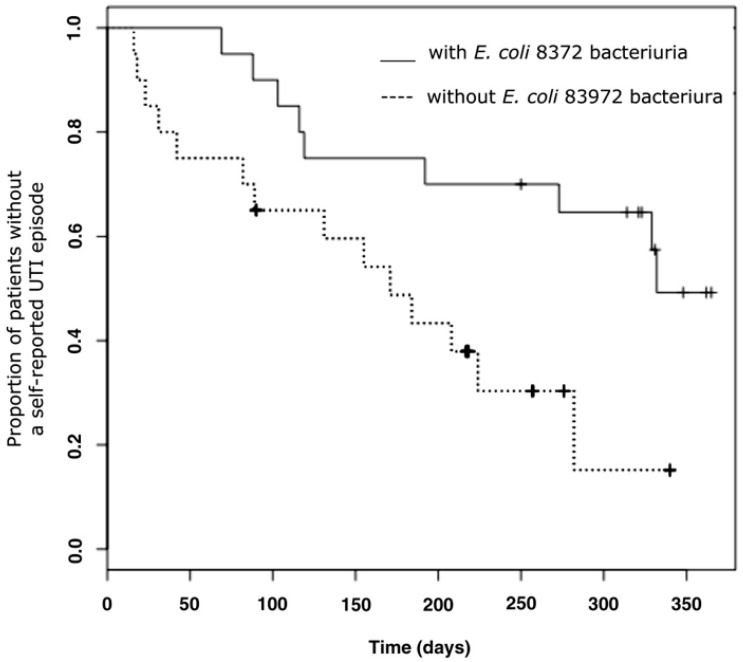
Protection by *E. coli* 83972 bacteriuria compared to the placebo arm of the study. Risk for symptomatic UTI in 20 patients, who were randomized to blinded *E. coli* 83972 inoculations or placebo. After 12 months of observation, a cross-over was performed. The median time to the first symptomatic UTI was longer in patients with *E. coli* 83972 bacteriuria (median 11.3 vs. 5.7 months, sign test *p* = 0.0129). Published with permission from the Journal of Urology [16].

The time to first UTI was significantly longer in the group inoculated with *E. coli* 83972 (median 11 vs. six months, *p* = 0.0129), and the number of UTI episodes was significantly lower (13 vs. 35 UTI episodes, *p* = 0.009) in patients with *E. coli* 83972 bacteriuria compared to the placebo control-arm (Figure 2) [16]. There were no significant side effects or febrile UTI episodes registered during the study. These findings verify the protective effect of *E. coli* 83972 inoculation against symptomatic UTI.

## 3. Studies of Bacteria and Their Infected Hosts

This challenge model also makes it possible to ask molecular questions directly in human hosts. In addition to being therapeutic, the *E. coli* 83972 human inoculation protocol provides a unique research model for studies of host-parasite interaction in vivo in the human urinary tract. We have used this opportunity to study determinants of bacterial establishment in the lower urinary tract and have characterized the innate immune response of infected hosts.

### 3.1. The Role of the P and Type 1 Fimbriae

*E. coli* 83972 establishes bacteriuria in patients with incomplete bladder emptying [19], but so far, the establishment of bacteruria has been unsuccessful in patients with intact bladder voiding. In an attempt to extend the inoculation therapy to patients with recurrent UTI and complete bladder emptying, we have chosen to construct variants of *E. coli* 83972, which express functional fimbriae. P fimbriae and type 1 fimbriae were selected as *E. coli* 83972 carries defective copies of these genes [20,31], and as extensive published data suggests that fimbriae enhance bacterial fitness for the urinary tract in animal models and clinical studies [36,37,38,39,40].

#### 3.1.1. Fimbriated Transformants of *E. coli* 83972

To study the role of *E. coli* adhesion in human UTI pathogenesis and to enhance the colonization fitness of *E. coli* 83972, we designed a study protocol in which patients were inoculated with transformants of *E. coli* 83972, carrying plasmids with functional copies of the *pap/prs* or *fim* gene clusters (encoding P or Type 1 fimbriae) [33,40,41]. P-fimbriated *E. coli* 83972 expressed functional fimbriae *in vivo*, as shown by adherence to exfoliated epithelial cells in the urine of colonized patients [40] and agglutination of human erythrocytes in vitro [33].

Patients were inoculated sequentially, with wild-type *E. coli* 83972, and the P- or Type 1–fimbriated variants. Bacterial persistence and host immune activation was compared, intra-individually, to reduce the influence of host-specific variation. Importantly, patients remained asymptomatic while carrying the P-fimbriated *E. coli* 83972 transformant and the Type 1–fimbriated variant.

The P-fimbriated transformants established bacteriuria more rapidly and required a lower number of bacteria to reach significant numbers [33]. The P-fimbriated transformants triggered higher levels of urine cytokines such as IL-6 and IL-8 and a higher neutrophil response [41]. P fimbrial expression was lost over time, due to plasmid segregation.

To directly evaluate the effect of adhesion, inoculations were performed with a tranformant lacking the P fimbrial adhesin PapG. The PapG-negative strain did not show a competitive advantage compared to the wild-type strain, confirming the role of the adhesin. IL-6, IL-8 and PMN infiltration remained at the same levels when compared to inoculation with the wild-type strain [42].

In contrast, the Type 1–fimbriated transformant did not change the rate of bacterial establishment or trigger inflammation in the human urinary tract [42]. In addition, the Type 1–fimbriated transformants adhered poorly to exfoliated human uroepithelial cells [42].

#### 3.1.2. Reconstituted Chromosomal Fimbrial Gene Clusters

In a recent study, we used *E. coli* 83972 variants with reconstituted chromosomal *pap* or *fim* gene clusters [43]. The strains express functional P fimbriae or Type 1 fimbriae. Five patients were inoculated with the chromosomally transformed *fim* and *pap* variants on different occasions, as well as *E. coli 83972*. The host response to inoculation was followed by repeated blood samples for transcriptomic analysis and urine sampling to measure the concentration of inflammatory mediators. Data derived from the transcriptomic analysis demonstrated that P fimbriae reprogram host gene expression, for example Type I IFN genes and TLR-dependent innate immune activation pathways. Importantly, P and Type 1 fimbriae activated distinctly different pathways within the host, detectable as early as three hours post-inoculation [43].

### 3.2. Host Response to *E. coli* 83972 in Inoculated Human Hosts

To characterize the innate immune response in the urinary tract, we examined the urine proteome in patients inoculated with *E. coli* 83972. The urine sampling was performed monthly during the prospective study of Sundén et al. [16] and was repeated when some patients were subjected to a second or a third inoculation [44].

An increase in urine neutrophil numbers and IL-8 concentrations was observed in several patients, compared to pre-inoculation samples obtained under sterile conditions. In contrast, IL-6, which is produced during acute pyelonephritis, was not affected. Based on repeated inoculations in some patients who lost the ABU strain, we observed that the host response pattern is reproducible in individual patients [44].

### 3.3. Genetic Control of the Innate Immune Response during *E. coli* 83972 Inoculation

The innate immune response to UTI is controlled by TLR4 and genes in the signaling pathway (see Svanborg et al., this issue). Mice lacking *Tlr4*, *MyD88*, *Trif* or *Tram* develop ABU due to an aborted innate immune response. In contrast, mice lacking the downstream effector genes such as *Irf3*, which controls the antibacterial defense, may develop severe acute pyelonephritis and renal scarring. In UTI-prone patients, gene deletions are rarely seen, but promoter polymorphisms have been identified. Promoter variants that reduce TLR4 expression are associated with ABU while promoters affecting *IRF3* expression are found in patients with APN.

To address if the innate immune response to ABU is genetically determined, we genotyped patients carrying *E. coli* 83972 for the ABU-associated TLR4 promoter polymorphisms and IRF3 promoter polymorphisms [45,46]. Low neutrophil, IL-6, IP-10, MCP-1 and sIL2Rα concentrations were found in patients with ABU-associated TLR4 promoter polymorphisms. Patients carrying the ABU-associated, heterozygous (–925, –776, A/G–C/T) IRF3 promoter genotype had significantly lower urine neutrophil numbers and IL-6 and MCP-1 concentrations in urine than the inoculated patients without the ABU-associated genotypes [44].

The results identify a low but detectable innate immune response to *E. coli* 83972 ABU, with individual variation associated with *TLR4* and *IRF3* polymorphisms.

## 4. Assessment of Risk that *E. coli* 83972 Regains Virulence in the Human Urinary Tract

Despite the extensive analysis of genome attenuation in *E. coli* 83972, the relatedness of *E. coli* 83972 to uropathogenic *E. coli* strains might raise the question if *E. coli* 83972 can regain virulence during growth in the human host. This was addressed in a paper by Köves et al. [47], where we examined isolates from the few patients in the prospective study who experienced symptoms at some time point during *E. coli* 83972 carriage.

Four patients developed symptoms during *E. coli* 83972 bacteriuria. The *fim*, *pap*, *foc, hlyA*, *fyuA*, *iuc*, *iroN*, *kpsMT* K5 and *malX* genotypes of the symptomatic reisolates remained unchanged. Bacterial gene expression profiles were unique for each host, providing no evidence for common de-regulation. The symptomatic isolates did not differ in virulence from the wild-type strain, as defined in the murine UTI model by persistence, symptoms or innate immune activation. We concluded that the switch from asymptomatic *E. coli* 83972 carriage to symptomatic UTI was not explained by a reversion to a functional virulence gene repertoire.

## 5. Conclusions

*E. coli* 83972 inoculation establishes protective ABU in patients with complicated recurrent UTI and reduces the frequency of symptomatic UTI in this patient group. The protocol is safe and efficient, as defined in a placebo-controlled trial. The strain *E. coli* 83972, also called “the nice bug’’, is freely available and we are happy to assist those who are interested in setting up this therapeutic protocol in their research centers. The inoculation model also provides, by add-on protocols, a unique research model of bacteria-host interaction, where the effect of the genetic modification of virulence and/or adherence properties of *E. coli* 83972 can be studied in vivo in the human urinary tract.

## Appendix

All our studies reviewed in this manuscript have been approved by the Ethical Committee, Lund University (LU-32-00, LU-742-01 and 2010/463).
